# Severe Headache with Eye Involvement from Herpes Zoster Ophthalmicus, Trigeminal Tract, and Brainstem Nuclei

**DOI:** 10.1155/2015/402015

**Published:** 2015-04-02

**Authors:** Sasitorn Siritho, Wadchara Pumpradit, Wiboon Suriyajakryuththana, Krit Pongpirul

**Affiliations:** ^1^Bumrungrad International Hospital, Bangkok 10110, Thailand; ^2^Department of Preventive and Social Medicine, Faculty of Medicine, Chulalongkorn University, Bangkok 10330, Thailand; ^3^Department of International Health, Johns Hopkins Bloomberg School of Public Health, Baltimore, MD 21205, USA

## Abstract

A 43-year-old female presented with severe sharp stabbing right-sided periorbital and retroorbital area headache, dull-aching unilateral jaw pain, eyelid swelling, ptosis, and tearing of the right eye but no rash. The pain episodes lasted five minutes to one hour and occurred 10–15 times per day with unremitting milder pain between the attacks. She later developed an erythematous maculopapular rash over the right forehead and therefore was treated with antivirals. MRI performed one month after the onset revealed small hypersignal-T2 in the right dorsolateral mid-pons and from the right dorsolateral aspect of the pontomedullary region to the right dorsolateral aspect of the upper cervical cord, along the course of the principal sensory nucleus and spinal nucleus of the right trigeminal nerve. No definite contrast enhancement of the right brain stem/upper cervical cord was seen. Orbital imaging showed no abnormality of bilateral optic nerves/chiasm, extraocular muscles, and globes. Slight enhancement of the right V1, V2, and the cisterna right trigeminal nerve was detected. Our findings support the hypothesis of direct involvement by virus theory, reflecting rostral viral transmission along the gasserian ganglion to the trigeminal nuclei at brainstem and caudal spreading along the descending tract of CN V.

A 43-year-old Canadian female presented with a two-day history of severe stabbing right-sided periorbital and retroorbital area headache, along with dull-aching unilateral jaw pain. The pain was initially described as “pins and needles” but subsequently became stabbing in nature. The pain episodes lasted five minutes to one hour and occurred 10–15 times per day with unremitting milder pain between the attacks. She also had eyelid swelling, ptosis, and tearing of the right eye but no rash. She had history of chicken pox in childhood.

An initial brain MRI was unremarkable and she was diagnosed with trigeminal cephalalgia and treated symptomatically. Two weeks later, she returned with persistent but somewhat improved pain. Her autonomic symptoms, photophobia, tearing, and eyelid swelling over the right eye with no corneal involvement were noted along with rash without vesicle over her nose. Oral valacyclovir, topical acyclovir, and antibiotics were prescribed. She returned six days later with severe pain and possible keratitis and was transferred to our hospital.

Physical examination revealed maculopapular rash in the right forehead without typical vesicles over right periorbital area, keratitis, photophobia, tearing, mildly dilated pupil with no reaction to light with visual acuity of 20/40, normal fundus, minimal eyelid drooping, decreased corneal reflex, and a mild cellular infiltrate in the anterior chamber of the right side. No proptosis was observed. She complained of electrical shock-like pain involving her neck, right cheek, and right periorbital and retroorbital area and jaw. No temporal artery tenderness or nodularity, no weakness of muscles of mastication, and no definite motor weakness were seen elsewhere. The diagnosis of Herpes Zoster Ophthalmicus with uveitis was made.

Because of her severe headache and pain, involving multiple cranial nerves, the patient underwent MRI of the brain and orbit one month after the onset, to exclude spreading infection, which revealed small hypersignal-T2 in the right dorsolateral mid-pons and from the right dorsolateral aspect of the pontomedullary region to the right dorsolateral aspect of the upper cervical cord, along the course of the principal sensory nucleus and spinal nucleus of the right trigeminal nerve. No definite contrast enhancement of the right brain stem/upper cervical cord was seen. Orbital imaging showed no abnormal signal intensity or definite enhancement of bilateral optic nerves/chiasm, extraocular muscles, and globes. Slight enhancement of the right V1, V2, and the cisterna right trigeminal nerve was detected (Figures 1 and 2).

She was given intravenous and topical acyclovir for three weeks, along with antibiotics and other medications for postherpetic neuralgia. Her headache and eye symptoms responded dramatically to the treatment; the skin of the right cheek became less severely hypersensitive. Brain MRI showed a slight decrease in the enhancement of the lesions previously seen.

Dermatomal pain with eye involvement without typical papulovesicular skin lesion was suggestive of Ophthalmic Zoster Sine Herpete [1], which can be accompanied by signs and symptoms of CN III paresis [2]. In this case, patient had been partially treated with antivirals prior to the rash that appeared so this may conceal her symptoms and signs as well as MRI enhancement.

Herpetic involvement of cranial nerves has been most likely underestimated. Even more difficult to find is imaging evidence that demonstrates the CN V connection pathway to the nuclear lesion at brainstem (rhombencephalitis) [3–5].

Similar to our findings, M. Aribandi and L. Aribandi demonstrated abnormal hypersignal in the left lower pons and medulla posteriorly and laterally near the fourth ventricle, extending into the upper cervical cord, corresponding to the location of the spinal trigeminal nucleus and tract [3]. Haanpää et al. described focal nonenhancing brainstem hyperintense lesions in T2-weighted images in patients with trigeminal zoster without extraocular muscle involvement [4]. Our MRI findings demonstrated an even longer spreading of the abnormal hyperintensity signal on T2W compared to a case report by Tsai et al. which described a long enhancing lesion from upper cervical cord extending to the lower thoracic spinal cord [6].

Interestingly we found ptosis and pupillary dilatation, which might be from the involvement of the third nerve. Similar to our study, Hu et al. reported a patient with brainstem and multiple cranial nerves involvement including ptosis but no pupillary involvement in immunocompetent host [7]. Our MRI findings may support the hypothesis of direct involvement by virus that VZV migrated transaxonally or transynaptically into the brain stem parenchyma and may spread to multiple cranial nerve, reflecting rostral viral transmission along the gasserian ganglion to the trigeminal nuclei at brainstem and caudal spreading along the descending tract of CN V.

## Figures and Tables

**Figure 1:**
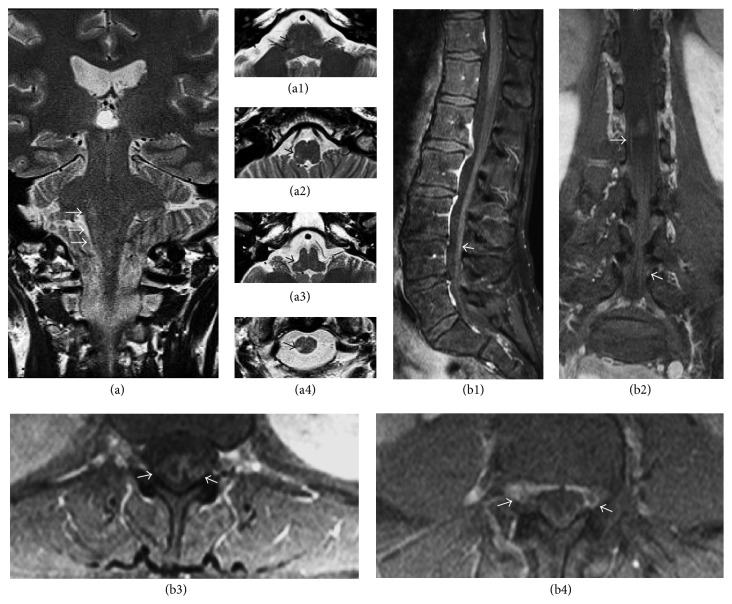
(a) Coronal and axial MRI sections of the brainstem to spinal cord showed small hypersignal-T2 in the right dorsolateral mid-pons (a1) and from the right dorsolateral aspect of the pontomedullary region (a2) to the right dorsolateral aspect of the upper cervical cord (a3-a4), along the course of the principle sensory nucleus and spinal nucleus of the right trigeminal nerve. Sagittal (b1), coronal (b2), and axial (b3-b4) spinal MRI showed enhancement of the nerve roots along the spinal cord.

**Figure 2:**
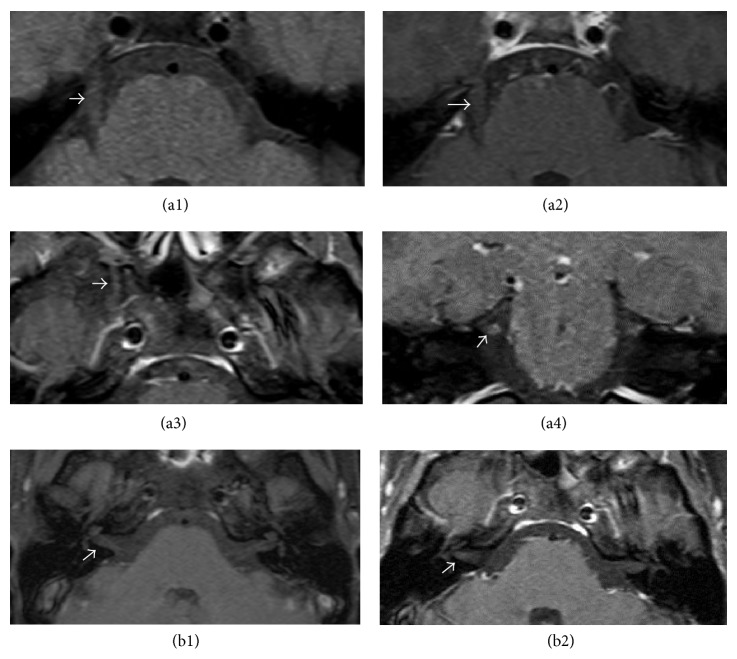
Axial MRI sections of the brainstem at the pontine level demonstrated fusiform enlargement of the fifth cranial nerve at the cisterna level on the T1-weighted images (a1). Postcontrast, fat-suppressed T1 images showed slight enhancement that is disclosed on cisterna level of the fifth cranial nerve (a2) and enlargement of the maxillary division of the fifth cranial nerve with slight enhancement while it passed foramen rotundum (a3). Also coronal MRI section at the level of the fifth cranial nerve showed slight enhancement (a4). Axial MRI sections showed brain stem at the level of facial and vestibulocochlear nerves in precontrast image (b1) and slight enhancement in postcontrast study (b2).
